# Lipid-anchored melanotransferrin mediates transferrin-independent iron uptake and ferritin storage in mammals

**DOI:** 10.1038/s41420-026-03043-9

**Published:** 2026-04-17

**Authors:** Mei Mei Tian, Jacqueline W. C. Tiong, Reinhard Gabathuler, Garnet Martens, Elaine C. Humphrey, Wilfred A. Jefferies

**Affiliations:** 1 The Michael Smith Laboratories, University of British Columbia, Vancouver, BC Canada; 2 Department of Microbiology and Immunology, University of British Columbia, Vancouver, BC Canada; 3 BioImaging Facility, University of British Columbia, Vancouver, BC Canada; 4 Department of Zoology, University of British Columbia, Vancouver, BC Canada; 5 Department of Medical Genetics, University of British Columbia, Vancouver, BC Canada; 6 Department of Urological Sciences, University of British Columbia, Vancouver, BC Canada; 7 Vancouver Prostate Centre, Vancouver Coastal Health Research Institute, Vancouver, BC Canada; 8 Centre for Blood Research, University of British Columbia, Vancouver, BC Canada; 9 The Djavad Mowafaghian Centre for Brain Health, University of British Columbia, Vancouver, BC Canada

**Keywords:** Endocytosis, Iron

## Abstract

Non-transferrin-bound iron (NTBI) transport constitutes a critical pathway for cellular iron uptake in the kingdom *Animalia* that remains mechanistically unresolved. Its physiological importance is underscored by atransferrinemia, a rare disorder in which individuals lacking plasma transferrin nonetheless retain the capacity to distribute dietary iron to essential organs, implying the presence of compensatory iron transport routes. Melanotransferrin (MFI2; also designated p97 or CD228) is an evolutionarily conserved iron-binding protein that exists in both a secreted form and a glycosylphosphatidylinositol (GPI)-anchored membrane-bound form, suggesting a fundamental role in iron homeostasis. In mammals, the secreted isoform mediates iron transport across the blood–brain barrier, whereas GPI-anchored MFI2 is expressed by microglia in proximity to β-amyloid plaques in Alzheimer’s disease, implicating it in neuroinflammatory processes. Moreover, it is also recognized as a tumor-associated antigen in melanoma, indicating a potential role in tumor progression. In the present study, we delineate a previously uncharacterized NTBI internalization pathway mediated by GPI-MFI2. Using human melanoma cells, we demonstrate that GPI-MFI2, together with its bound iron, undergoes caveolae-dependent internalization followed by trafficking through a Rab5-mediated endosomal pathway. The internalized iron is subsequently trafficked to ferritin, underscoring its functional importance in maintaining intracellular iron stores. These findings establish the first molecularly defined pathway for transferrin-independent iron uptake in mammalian cells, providing a framework to interrogate MFI2’s role in iron mobilization and dysregulation in neurodegeneration and cancer.

## Introduction

Iron is an indispensable trace element that sustains fundamental cellular functions, including mitochondrial respiration, DNA synthesis, and redox homeostasis. Landmark discoveries in iron biology, such as the molecular cloning of the divalent metal transporter DMT1 (Nramp2) [1, 2], the iron exporter ferroportin [3], transferrin receptor 2 [4], and iron-regulated ferric reductases [5], have substantially advanced our understanding of iron metabolism and systemic homeostasis. Nevertheless, the canonical transferrin/transferrin receptor (Tf/TfR) endocytosis pathway remains the most extensively characterized and physiologically dominant mechanism governing cellular iron uptake [6, 7]. Iron-loaded Tf binds to the TfR on the cell surface at physiological pH and is internalized through a clathrin-mediated endosomal pathway. The intra-vesicular pH causes a decrease in the affinity between iron and Tf, leading to the release of iron from the complex. The newly released iron is transported into the cytoplasm, mediated by a metal transporter known as Nramp2 [2, 8, 9]. The iron is either stored as ferritin or transported to other organelles for cellular usage [7, 10–12]. Structural studies examining the effect of iron binding on the human Tf/TfR complex show a conformational change in Tf, which hinders iron release from its high-affinity N-lobe and restricts accessibility to iron-accepting molecules [13]. One scenario suggested by this finding is that the atom of iron bound to the lower-affinity C-lobe is exclusively released during each cycle of internalization of the Tf/TfR complex [14]. However, Tf is not universally required for efficient iron uptake, as evident in hypotransferrinemic (hpx) mice, where massive amounts of iron accumulate in all non-hematopoietic tissues, even though their serum Tf levels are low [7, 15, 16]. The liver iron burden in hpx mice is 100-fold higher than that of the control, suggesting the existence of Tf/TfR-independent iron transport mechanisms [16–19]. These observations in hypotransferrinemic mice have prompted investigations into how cells acquire iron when Tf-mediated delivery is compromised or absent.

Recent studies have expanded our understanding of alternative cellular uptake mechanisms. Specifically, the internalization routes of glycosylphosphatidylinositol-anchored proteins (GPI-APs) have gained attention for their unique and diverse entry pathways into cells. Unlike transmembrane proteins, GPI-APs, are internalized through non-classical, clathrin-independent pathways, particularly via clathrin-independent carriers (CLICs) and GPI-AP-enriched early endosomal compartments (GEECs) [20–22]. These pathways are known to facilitate entry into distinct endosomal compartments, highlighting the unique endocytic behavior of GPI-APs. In addition, GPI-Aps have been shown to associate with lipid rafts, membrane microdomains rich in cholesterol and sphingolipids, that facilitate clustering and internalization [23, 24]. This interaction with lipid rafts underscores a complex mechanism that differs from classical endocytosis, further complicating our understanding of cellular uptake mechanisms. Interestingly, other findings suggest that GPI-APs may also be internalized via receptor-mediated pathways. For example, scavenger receptors, such as CD36 have been implicated in the uptake of GPI-anchored proteins in certain cell types [25]. Moreover, in immune cells, such as dendritic cells, GPI-APs undergo specialized internalization and trafficking processes that could influence antigen processing and presentation [21].

While the Tf/TfR pathway clearly plays a central role in iron homeostasis, emerging evidence suggests that cells may possess additional mechanisms for iron acquisition that operate independently of this classical route. Melanotransferrin (MFI2), is an iron-binding glycoprotein belonging to the transferrin family, which shares a 40% protein sequence identity with human lactoferrin [26]. Unlike other members of the Tf family, MFI2 can be found as either a membrane protein attached to the cell surface via a GPI-AP or as a soluble form in serum. The two forms originate from alternative splicing of the MFI2 mRNA [27]. Calorimetric studies show that MFI2 binds only one molecule of iron with an apparent binding affinity constant of 4.4 × 10¹⁷ M⁻¹, which is an affinity intermediate between the binding constants of iron to the N and C-lobe of Tf [28].

It is highly conserved across various species [26]. Using multi-sequence alignments and neighbor-joining trees of 71 transferrin family sequences from 51 different species, MFI2 has been proposed to be one of the oldest members of the transferrin family, dating back to more than 670 million years ago [29]. It emerged at the time of the divergence of *Protostomes* and *Deuterostomes*. Thus, MFI2 may have diverged from serum Tf soon after duplication of the iron-binding lobes [29]. In contrast to its ancient origin, MFI2 was first identified as the melanoma antigen MFI2, a cell-surface marker for human skin cancer. Subsequently, the protein was found to be expressed at various levels in the liver, intestine, umbilical cord, placenta, sweat gland, human brain endothelium, and more recently as a differentiation marker on chondrocytes and osteoblasts [7, 30].

Our previous research has shown that secreted soluble human MFI2 (sMFI2) is able to pass through the blood-brain barrier (BBB) into the brain, presumably by a receptor-mediated mechanism, while in comparison, Tf is unable to distribute to the brain parenchyma [7, 31]. Prior work by Smith and colleagues reported co-localization of GPI-linked MFI2 with caveolin-1 in melanoma cells in the context of investigating antibody-drug conjugate resistance mechanisms, though the functional significance of this association for iron uptake and transport was not established [32].

Paradoxically, findings suggest that MFI2 may function as a tumor suppressor in malignant melanoma [33]. CRISPR/Cas9-mediated knockout of MFI2 in human melanoma cells resulted in enhanced invasiveness and significant alterations in the expression of genes associated with tumorigenic progression and metastasis [33]. In vivo studies using severe combined immunodeficiency (SCID) mice further demonstrated increased tumor growth and metastatic potential of MFI2-deficient melanoma cells, indicating a previously unrecognized tumor-suppressive role for MFI2 [33]. Moreover, analysis of publicly available melanoma patient datasets revealed a positive correlation between MFI2 mRNA expression levels and overall patient survival, reinforcing its potential clinical relevance [33]. Similarly, Mazahreh et al. characterized SGN-CD228A, an MFI2-directed antibody–drug conjugate, demonstrating potent antitumor efficacy across diverse preclinical solid tumor models [34]. Furthermore, Zhang et al. reviewed MFI2’s broader physiological and pathological roles, highlighting its potential in targeted therapy and precision oncology [35]. Collectively, these findings challenge earlier reports describing MFI2 as a tumor-promoting factor and underscore the necessity for further investigation into its biological function and therapeutic potential in tumor management [33].

In addition, studies on Drosophila MFI2 have shown insect MFI2 to also be a GPI-linked, iron-binding protein attached to epithelial cell membranes. It is a septate junction component that forms the paracellular permeability barrier in epithelial tissues, and endocytosis and apicolateral recycling of iron-bound MFI2 facilitate septate junction assembly [36]. Moreover, Drosophila MFI2 mutants are genetically complemented by mouse MFI2 [36]. These diverse internalization routes and interactions suggest GPI- MFI2, may facilitate iron uptake across species in multiple cellular compartments, expanding the potential locations and molecular mechanisms involved in cellular iron homeostasis.

Overall, MFI2 provides a compelling non-Tf bound iron (NTBI) model to investigate TF/TFR-independent iron uptake, as it is evolutionarily conserved and in mammals its expression is markedly upregulated in specific pathological contexts where iron metabolism is dysregulated, including melanoma [37, 38], glioblastoma [35, 39], and Alzheimer’s disease [40–43]. The evolutionary conservation of MFI2 alongside the classical transferrin system suggests these proteins fulfill non-redundant physiological roles, yet why cells would maintain energetically costly parallel iron uptake mechanisms remains unclear. Furthermore, whether MFI2 internalization occurs through caveolae-dependent pathways and functionally contributes to cellular iron acquisition remained unresolved.

## Results

### GPI-MFI2 is endocytosed through caveolae

**Fig. 1:**
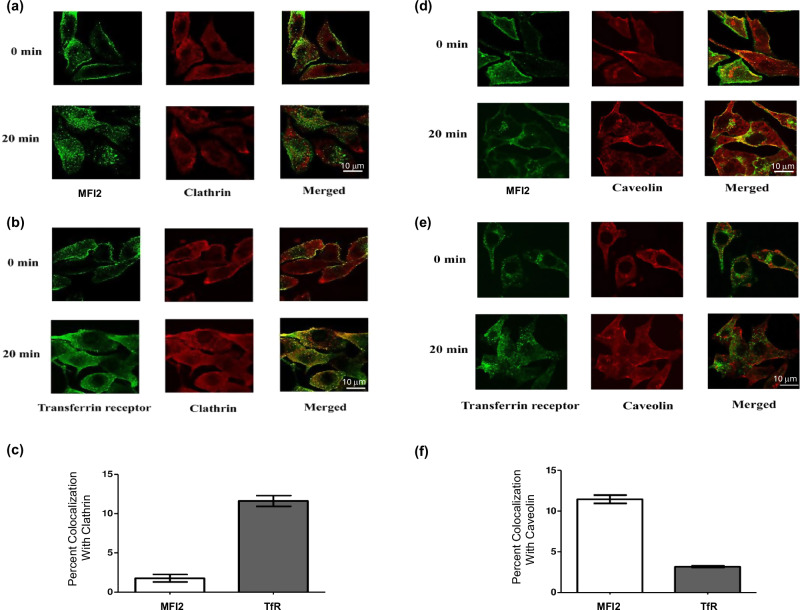
GPI-MFI2 co-localizes with caveolae not clathrin-coated pits in human SK-MEL 28 melanoma cells. Labeled GPI-MFI2 (antibody: L235) or TfR (antibody: OKT9) was visualized with Alexa 488-conjugated goat anti-mouse (green) and labeled caveolae or clathrin vesicles were visualized with Alexa 568-conjugated goat anti-rabbit antibodies (red). Merged images are shown on the right, and co-localization appear as yellow. Cells were fixed in 4% paraformaldehyde and 2% glutaraldehyde to prevent redistribution of GPI-anchored protein triggered by antibody cross-linking. The scale bar represents 10 μm. Quantification of co-localization was performed using Image J 1.37c. Data shown were compiled from at least 20 individual cells from two to three separate individual preparations. Error bars represent standard error of the mean (SEM). **a** Confocal micrographs showing GPI-MFI2 does not co-localize with clathrin. **b** Confocal micrographs TfR co-localizes with clathrin coated vesicles. **c** Quantification of co-localization of clathrin-coated vesicles with GPI-MFI2 and TfR. **d** Confocal micrographs showing that GPI-MFI2 co-localizes with caveolin-1. **e** Confocal micrographs showing that TfR does not co-localize with caveolin-1. **f** Quantification of co-localization of caveolae with GPI-MFI2 and TfR.

Though some features relating to the internalization of GPI-anchored proteins have been examined [44, 45] debate still exists concerning the mechanisms involved [46–48]. To determine whether GPI-MFI2 co-localizes with caveolae or clathrin-coated pits, we employed a modified internalization assay previously used in measuring the endocytosis of other membrane receptors [49]. MFI2 is highly expressed in melanoma and other malignancies [4, 38, 50] and SK-MEL 28 melanoma cells were selected as the experimental system due to their exceptionally high expression of MFI2 (among the highest reported levels [34]), making them optimal for studying MFI2-specific trafficking mechanisms without overwhelming background from other iron transporters. Briefly, surface TfR or GPI-MFI2 were labeled with anti-TfR and anti-MFI2 antibody at 4 °C to block endocytosis in human SK-MEL 28 melanoma cells. Cells were returned to 37 °C for various time periods to allow endocytosis to resume and internalization of the labeled surface proteins was visualized using immunofluorescence microscopy. Fixing reagent containing 2% glutaraldehyde and 4% paraformaldehyde was used to ensure that the results were not triggered by antibody cross-linking leading to the sequestration of GPI-anchored proteins in caveolae as proposed by Mayor et al. GPI-anchored proteins tend to redistribute under normal fixing conditions, however, this redistribution is prevented by 0.3–0.5% glutaraldehyde/3% paraformaldehyde fixation [51]. Immunofluorescence microscopy results show that GPI-MFI2 does not co-localize with clathrin-specific staining after 20 min of endocytosis (Fig. 1a), while GPI-MFI2 co-localizes with caveolin-1 during the endocytotic process (Fig. 1d). Conversely, TfR does co-localize with clathrin-specific staining (Fig. 1b), but does not co-localize significantly with caveolae vesicles (Fig. 1e). This demonstrates that GPI-MFI2 enters the cell via a clathrin-independent pathway, whereas TfR enters by a clathrin-dependent endocytosis pathway. The percent co-localization of either GPI-MFI2 or TfR with both clathrin (Fig. 1c) and caveolin (Fig. 1f) are shown as bar graphs, where the fluorescence overlaps were quantified using ImageJ.

**Fig. 2:**
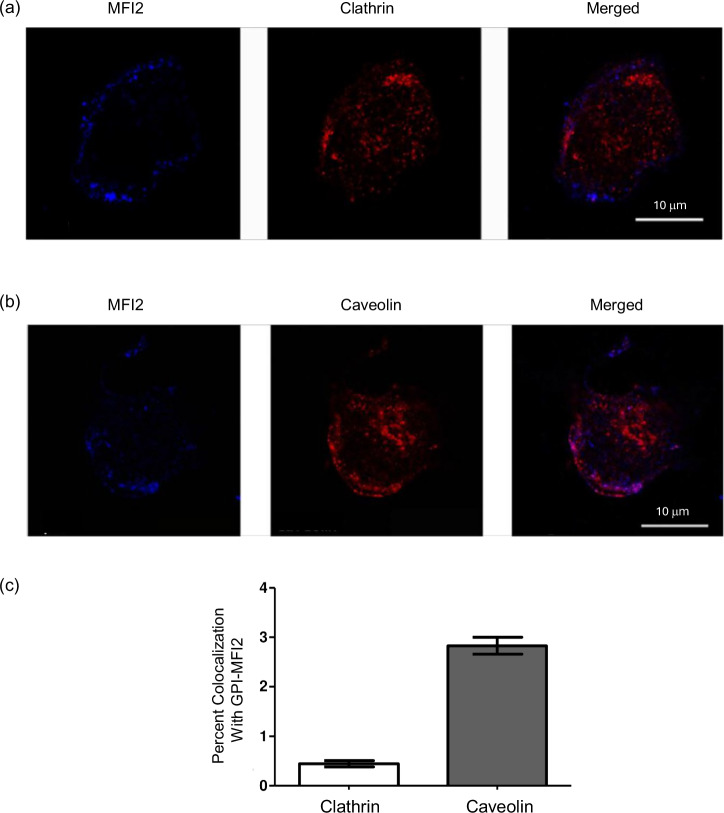
GPI-anchored MFI2 co-localize with caveolin, but not clathrin in human SK-MEL 28 melanoma cells. GPI-anchored MFI2 (blue) was labeled with FITC-Fab fragment made from monoclonal antibody against MFI2 (L235). Caveolae vesicles (red) were identified using caveolin- 1, while clathrin vesicles (red) were identified using an antibody against clathrin heavy chain. Both caveolae and clathrin vesicles were visualized with Alexa 568-conjugated goat anti-rabbit antibodies. Merged images are shown on the right, where colocalization (pink-purple) is shown. The scale bar represents 10 µm. Data shown represent a single plane at mid-section of the cell. **a** Colocalization of GPI-MFI2 with clathrin-coated pits. **b** Colocalization with GPI-MFI2 with caveolae. **c** Quantification of colocalization of clathrin or caveolae with GPI-MFI2. Co-localization was quantified using Image J 1.37c., where data shown were compiled from at least 20 individual cells from two to three separate individual preparations. Error bars represent ± SEM.

In order to rule out internalization due to Fc region mediated cross-linking, FITC-labeled Fab fragment made from the monoclonal antibody against GPI-MFI2 was used to confirm the co-localization between caveolin-1 and MFI2 (Fig. 2b), but not clathrin (Fig. 2a). Percent co-localization of GPI-MFI2 with both clathrin and caveolae is shown as a bar graph, after the fluorescence overlaps were quantified using ImageJ (Fig. 2c).

**Fig. 3:**
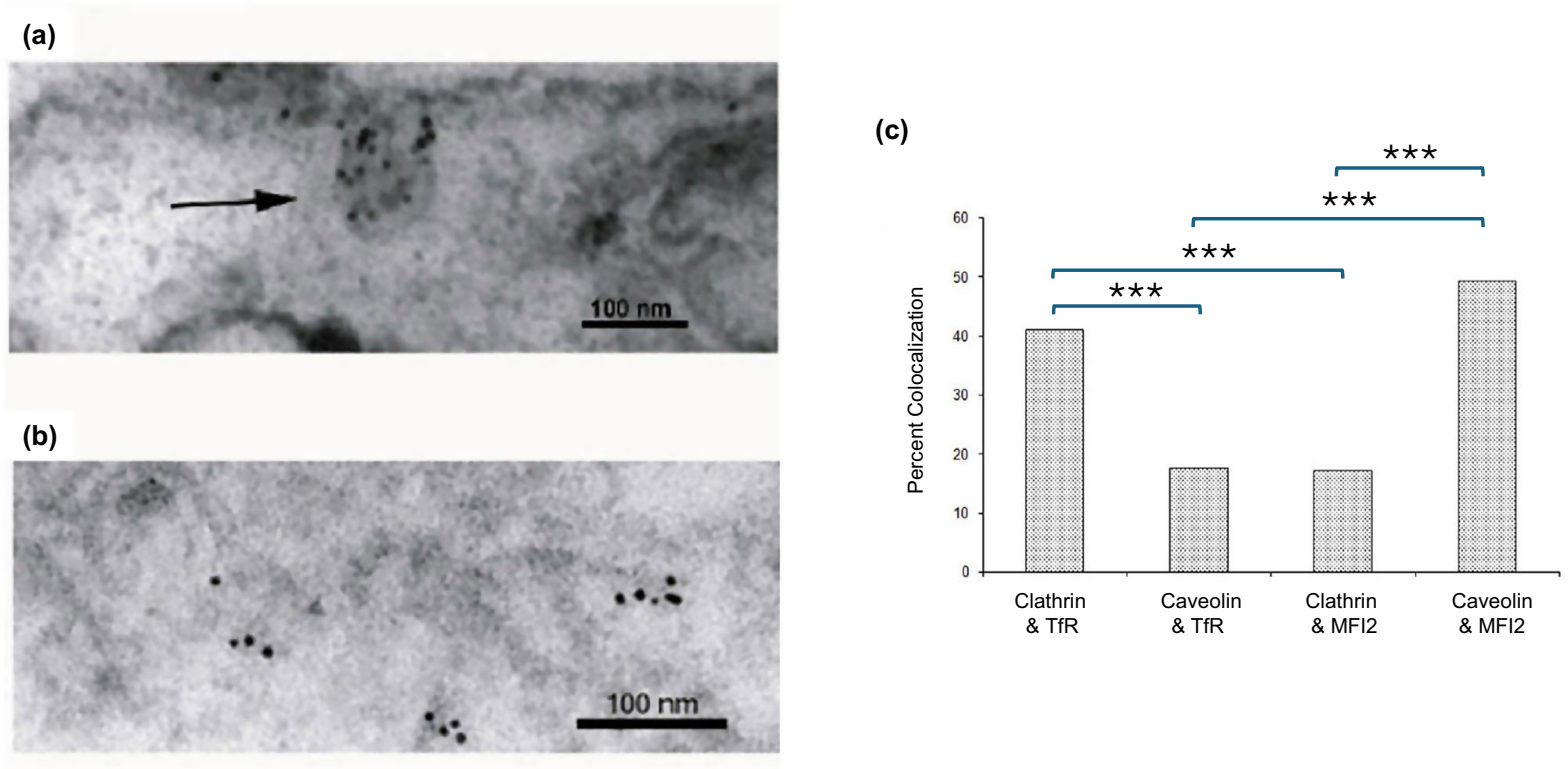
GPI-MFI2 localize in the same vesicle as caveolin-1, while TfR are localized in the clathrin coated vesicles in human SK-MEL 28 melanoma cells using immunoelectron microscopy. **a** Immunoelectron micrograph demonstrating the co-localization of caveolin-1 and GPI-MFI2 within the same vesicle (black arrow). **b** Immunoelectron micrograph demonstrating the co-localization of clathrin and transferrin receptor. Caveolin-1and Clathrin are identified by the 10 nm gold particles whereas GPI-MFI2 and TfR are directly conjugated with 5 nm gold particles. **c** The prevalence of MFI2 or TfR co-localization with either caveolae or clathrin-coated vesicles, where the immunoelectron microscopy image data was analyzed using Z-statistic test, which enabled the determination of the prevalence of co-localization between pMFI2/TfR and caveolin/clathrin. The prevalence of caveolin was significantly greater in MFI2-labeled vesicles when compared to the prevalence of caveolin in TfR-labeled vesicles or the prevalence of clathrin in MFI2-labeled vesicles (*** *p* < 0.001).

Co-localization of GPI-MFI2 within caveolin-1 positive vesicles was quantified by cryo-immunogold electron microscopy in SK-MEL 28 cells labeled with different size gold particles conjugated directly to primary antibodies against either GPI-MFI2 or TfR. GPI-MFI2 showed a high degree of co-localized with caveolin-1 in the vesicles (Fig. 3a), while TfR co-localized within clathrin-coated vesicles. (Fig. 3b). The z statistic comparing two proportions was calculated to determine the statistical significance of the prevalence of co-localization. The data indicated significantly greater prevalence of caveolin-1 rather than clathrin in MFI2 labeled vesicles (*p* < 0.001) (Fig. 3c). Taken together, these data show that GPI-MFI2 is found predominantly in caveolae where it co-localizes with caveolin-1.

### Iron uptake by GPI-MFI2 is associated with caveolae

**Fig. 4:**
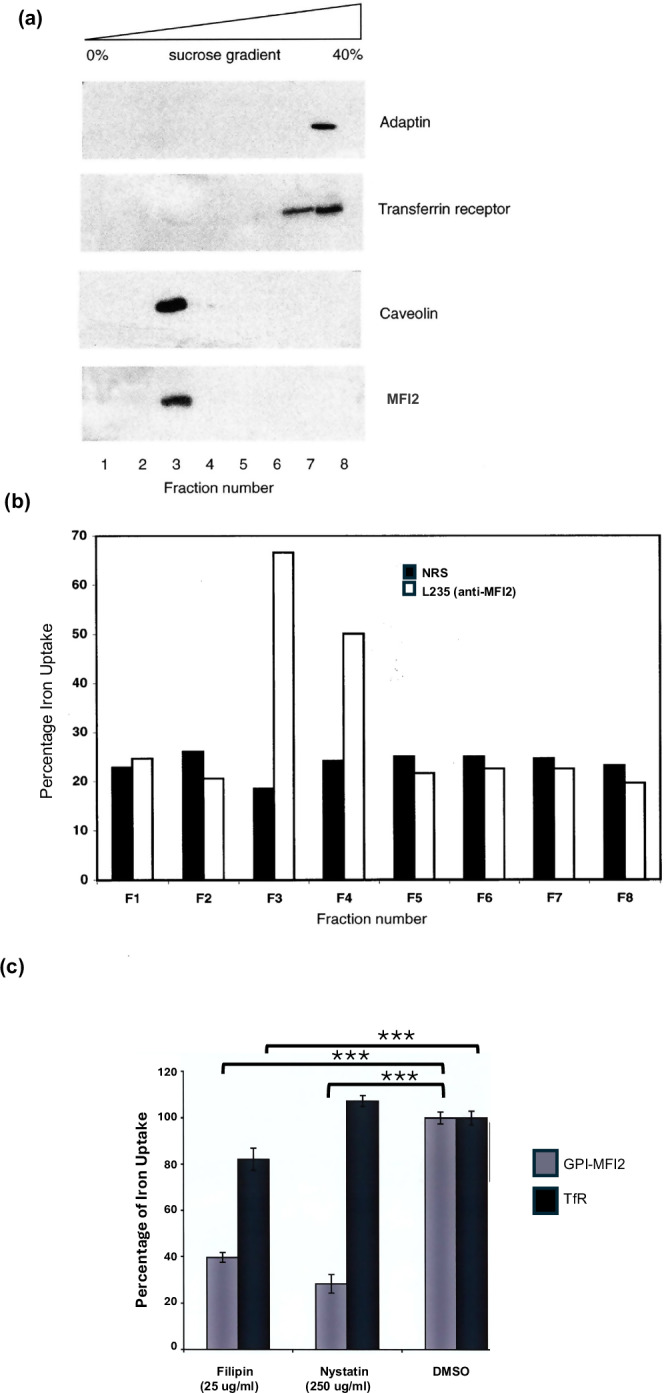
GPI-MFI2 is found in the same fraction as caveolae and mediates iron uptake in human SK-MEL 28 melanoma cells. **a** SK-MEL 28 cells were lysed on ice in 1% Triton X-100 in 140 mM KCl, 10 mM Tris-Cl, pH 7.5 and subjected to subcellular fractionation on a sucrose gradient. Eight fractions were collected and analyzed by SDS-PAGE and immunoblotted with antibodies against MFI2, Caveolin-1, TfR, and adaptin. **b** SK-MEL 28 cells were incubated with ^55^Fe-NTA prior to subcellular fractionation on sucrose gradient as in (**a**). Each of the eight fractions was immunoprecipitated for MFI2 using the antibody L235. As a control, normal rabbit serum (NRS) was used to immunoprecipitate the samples. The graph provides quantitative analysis of the ^55^Fe radioactivity detected from each fraction, representative of two separate experiments. **c** The presence of nystatin or filipin reduces the uptake of iron by GPI-MFI2, but has little or no effect on iron uptake through TfR. TRVb cell (TfR-negative CHO cells) that had been transfected with either MFI2 or TfR were treated with nystatin, filipin or the solvent DMSO before 1 μM of ⁵⁵Fe-NTA or 1 mg/ml of ⁵⁵Fe-Tf was added to the cells respectively. After 90 min, the cells were lysed and the radioactivity was measured. Data represent mean ± SEM from three independent experiments (*n* = 3). Statistical significance was determined by one-way ANOVA with Dunnett’s post-hoc test comparing each treatment to vehicle control. ***p < 0.001.

To provide independent biochemical evidence that GPI-MFI2 associate with caveolae, SK-MEL 28 cell lysates, fractionated by sucrose density centrifugation, were analyzed. Caveolae are insoluble in nonionic detergents, such as Triton X-100 at 4°C due to their unique lipid composition [52]. GPI-anchored proteins, cholesterol, glycosphingolipids and sphingomyelin can form a detergent-insoluble membrane domain in light buoyant density following a sucrose density centrifugation [48, 53]. The protein components within the fractions were identified by Western blot analysis. Both GPI-MFI2 and caveolin-1 were detected in fraction 3, whereas β-adaptin, a protein subunit of the clathrin complex, was detected in fraction 8. TfR, which utilize clathrin-dependent endocytosis, was detected in fractions 7 and 8 (Fig. 4a and Supplemental Data). To determine the ability of GPI-MFI2 to transport iron into the caveolae fraction, SK-MEL 28 cells were incubated with ^55^Fe-NTA at 37 °C prior to subcellular fractionation as described above. Each fraction was immunoprecipitated with antibodies against MFI2 or normal rabbit serum (control) and the associated radioactivity was measured. Figure 4b demonstrated that the majority of the ^55^Fe associates with GPI-MFI2 found in fraction 3 (Fig. 4b), which corresponds to the caveolae fraction found in the sucrose density gradient (Fig. 4a). In contrast, no radioactivity was detected in fraction 8 where TfR/β-adaptin was found (Fig. 4a,b).

Filipin and nystatin are cholesterol-binding drugs that are known to disrupt the structure of caveolae [54, 55]. Filipin is a polyene macrolide antibiotic that binds to cholesterol and disrupt the organization of the surrounding membrane [56–58]. Nystatin binds to cholesterol and is known to inhibit caveolae-dependent endocytosis [59–61]. TRVb cells, a CHO cell line with defective endogenous hamster TfR, transfected with either GPI-MFI2 or TfR were treated with filipin or nystatin. Filipin treatment of TRVb transfected cells under serum-free conditions reduced the uptake of ^55^Fe-citrate by 60%, while nystatin reduces the uptake of ^55^Fe-citrate by 70% (Fig. 4c). In contrast, filipin only reduced the uptake of ^55^Fe-Tf by 20%, whereas nystatin had little or no effect in the uptake of ^55^Fe-Tf (Fig. 4c). Therefore, the presence of cholesterol-binding reagents clearly affects the iron uptake by GPI-MFI2. Together with the previous results, we have demonstrated that caveolae mediate iron internalization via GPI-MFI2.

### GPI-MFI2 traffics to endosomes after internalization at a slower rate than TfR

**Fig. 5:**
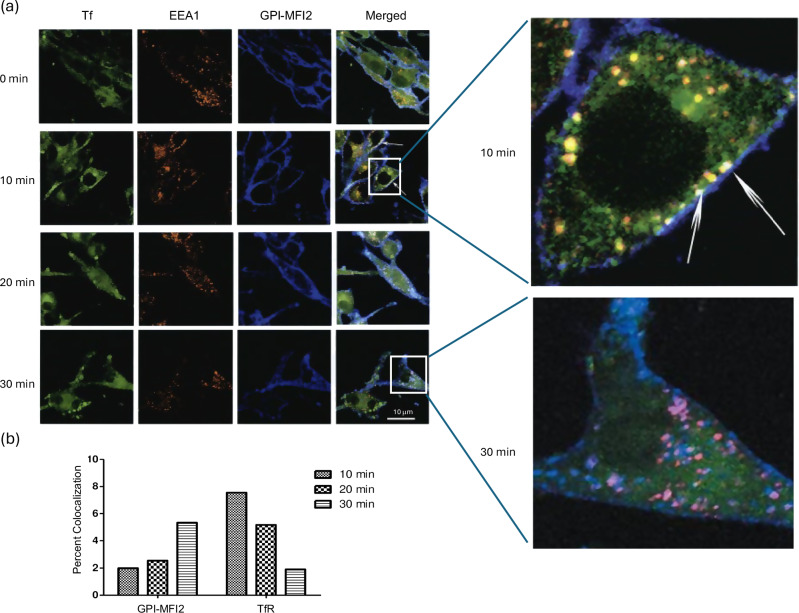
Intracellular trafficking of GPI-MFI2 involves early endosomes and GPI-MFI2 traffics at a slower rate than Tf in human SK-MEL 28 melanoma cells. **a** Cells were labeled with Alexa 488-conjugated Tf antibodies before the initiation of internalization for various periods of time at 37°C, fixed, permeabilized and stained for EEA1. Labeled GPI-MFI2 were visualized with Cy5-conjugated rabbit anti-mouse and labeled EEA1 were visualized with Alexa 568-conjugated rabbit anti-goat antibodies. Merged images are shown on the right. Tf =green; EEA1=red; GPI-MFI2=blue; Tf+EEA1=yellow; GPI-MFI2 + EEA=pink; Tf+EEA1 + GPI-MFI2=white. At 10 min, internalized Tf and EEA1 can be see merged (yellow), while MFI2 remains located close to the surface, with only a few spots of MFI2 with both Tf and EEA1 (white). At 30 min, internalized MFI2 can be seen located throughout the cytoplasm localized with EEA1 (pink) or Tf and EEA1 (white). These confocal micrographs are representatives of three separate experiments (*n* = 3); data shown represent a single plane at mid-section of the cell. The scale bar represents 10 µm. **b** Quantification of MFI2 and TfR colocalization with EEA-1 at various time points. Co-localizations were quantified using ImageJ 1.37c from the representative images shown in (**a**).

The time course of internalization and entry into the endosomal pathway of GPI-MFI2 was investigated in SK-MEL 28 cells, triple labeled for TfR, MFI2 and the early endosome marker, early endosome antigen-1 (EEA-1). At the initiation of the time course, both MFI2 and TfR were present at the cell surface of SK-MEL 28 whereas EEA-1 exhibited a typical punctate endosomal staining (Fig. 5a). After 10 min, most of the MFI2 molecules were still present on the cell surface whereas most of the TfR were endocytosed into the cells and co-localized with endosomes. A small fraction of the GPI-MFI2 molecules appear in the endosomes containing TfR, represented by the white punctate staining when the three immunofluorescence dyes (Alexa-488, Alexa-568 and Cy5) co-localized. This shows that the majority of GPI-MFI2 traffics more slowly to the endosomes than TfR. After 30 minutes of endocytosis, there is an increase in the number of MFI2 molecules associated with endosomes as shown by increase in the pink and white colocalization colors enlarged image in Fig. 5a. After 30 min, there are a significant fraction of endosomal vesicles containing MFI2 (Fig. 5a, b). The results show that GPI-MFI2 traffics to the endosomal compartment at a slower rate than the Tf/TfR complex (Fig. 5b).

### GPI-MFI2 donates internalized iron to ferritin

**Fig. 6:**
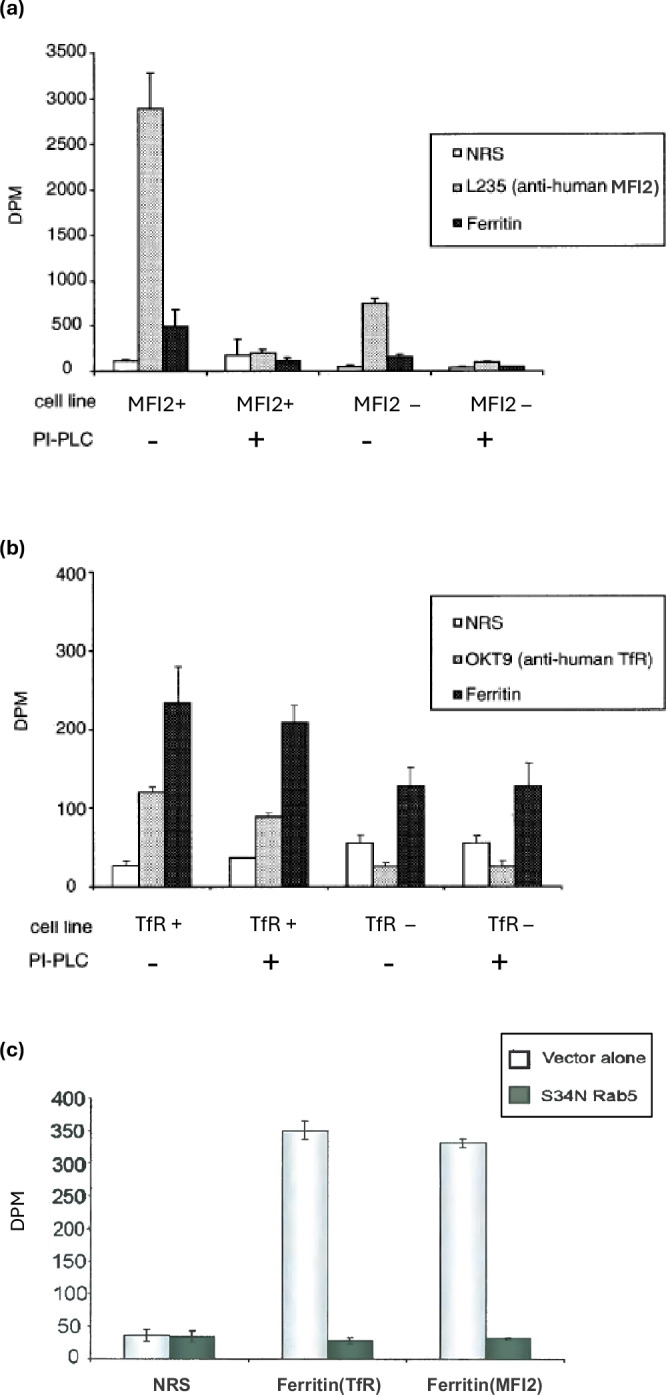
GPI-anchored MFI2 is able to donate iron to ferritin. Endogenous transferrin receptor-negative TRVb cells, and those transfected with human MFI2 (MFI2+TRVb) and human TfR (TfR+TRVb) were treated either with or without PI-PLC before they were incubated with **a** ^55^Fe-NTA for the MFI2+TRVb cells or **b** ^55^Fe-NTA loaded human transferrin for the TfR+TRVb cells. Antibodies against ferritin (DAKO), MFI2 (L235) (for the MFI2+TRVb cells) or TfR (OKT9) (for the TfR+TRVb cells) were used, with normal rabbit serum used to immunoprecipitate samples as the negative control. The radioactivity count associated with the immunoprecipitates was measured to evaluate iron delivery efficiency. The error bars represent the ± SD of three separate experiments. Statistical significance was determined by Student’s t-test (two-tailed, unpaired) comparing PI-PLC treated to untreated for each condition. *p < 0.05, **p < 0.01. **c** Endosomal disruption by dominant negative Rab5 (Rab5:S34N) prevents iron uptake mediated by either GPI-MFI2 or TfR in human SK-MEL 28 melanoma cells. Cells transfected with Rab5:S34N and vector alone were pre-starved for iron for one hour and incubated with 1 μM of ⁵⁵Fe-NTA for 90 minutes before the cells were lysed. Ferritin, MFI2 and TfR were immunoprecipitated from the lysate and the radioactivity was determined. Data represent mean ± SD from three independent experiments (*n* = 3). Statistical significance was determined by Student’s t-test (two-tailed, unpaired) comparing Rab5:S34N to vector control for each condition. **p < 0.01, ***p < 0.001.

To address whether GPI-MFI2 is capable of the incorporating internalized iron into ferritin on its own, we isolated ferritin by immunoprecipitation from TfR-deficient CHO cells (TRVb) or MFI2-transfected TRVb cells (TRVb+MFI2) incubated with ^55^Fe-NTA. These cells were treated with or without PI-PLC. When TRVb+MFI2 cells are treated with PI-PLC, the amount of ^55^Fe incorporated into ferritin decreases (Fig. 6a). Note that TRVb cells produce low levels of hamster MFI2, therefore we see some radioactive signal from L235-immunopreciptated cells which have not been transfected with MFI2, however this disappears upon treatment with PI-PLC. As a control, using TRVb cells or TfR-transfected TRVb cells, there was no significant difference in ^55^Fe incorporation of ferritin between the PI-PLC treated or non-treated cells as TfR are not sensitive to PI-PLC treatment (Fig. 6b). These results demonstrate that iron uptake mediated by GPI-MFI2 is able to transport iron from cell surface and donate to ferritin for storage.

### Disruption of the endosomal pathway significantly reduces the iron uptake mediated by GPI-MFI2

Rab5 is a small GTPase that regulates the fusion between endocytic vesicles and early endosome, as well as the homotypic fusion among the early endosomes [62]. A dominant negative rab5 (S34N rab5) mutation completely eliminates the GTP binding ability of rab5 and causes a dominant inhibitory effect over endocytosis at the level of endosomes fusion [63, 64]. The disruption of the endosomal pathway by the S34N mutation was used to study the effect of GPI-MFI2 and TfR on cellular iron uptake. Immunoprecipitation of ferritin from rab5:S34N transfected SK-MEL 28 cells incubated with^55^Fe-NTA under Tf-free conditions led to dramatic reductions in the amount of radioactivity associated with ferritin as compared to vector control transfectants (Fig. 6c). A similar reduction in the amount of radioactivity associated with ferritin was observed with rab5:S34N transfected SK-MEL 28 cells incubated in ^55^Fe-Tf under phosphatidylinositol-specific phospholipase C (PI-PLC) treatment (Fig. 6c). PI-PLC is an enzyme used to cleave GPI-anchored proteins off the cell surface to ensure that the iron loading of ferritin in these cells is solely dependent on Tf/TfR pathway. The disruption of endosomal pathway is sufficient to abolish iron uptake from either the Tf/TfR or the GPI-MFI2 pathways, thus confirming that endosomes are required for the iron transport mediated by GPI-MFI2 to ferritin.

**Fig. 7:**
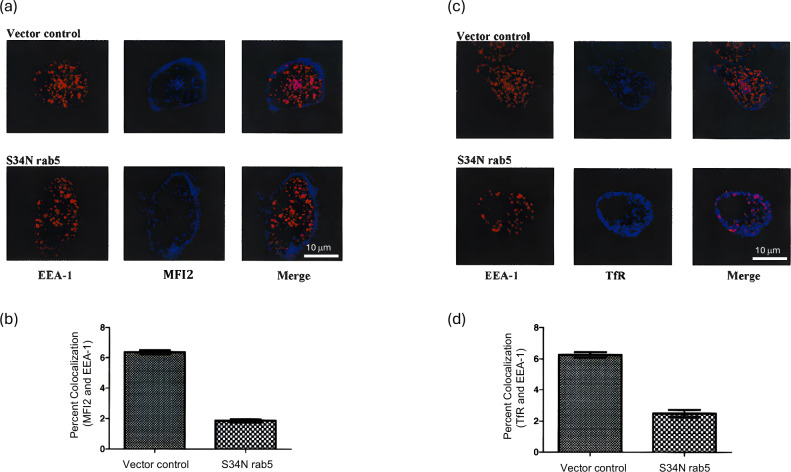
Endosomes are essential for GPI-anchored MFI2 internalization in human SK-MEL 28 melanoma cells. SK-MEL 28 cells were transfected with either vector control or S34N rab5 dominant negative vector. GPI-anchored MFI2 was labeled with monoclonal antibody against p97 (L235; blue), TfR was labeled with OKT9 antibody (blue) and early endosomes were identified using EEA- 1 (red). The scale bar represents 10 µm. Data shown represent a single plane at mid-section of the cell. Co-localization data shown were compiled from at least 20 individual cells from two to three separate individual preparations, and error bars represent ± SEM. **a** Fluorescent confocal images of cells showing co-localization between MFI2 and EEA- 1. **b** Percentage co-localization between MFI2 and EEA-1 is shown graphically. Co-localization (pink) was quantified using ImageJ 1.37c. **c** Fluorescent confocal images of cells showing co-localization between TfR and EEA-1. **d** Percentage co-localization between TfR and EEA-1 is shown graphically. Co-localization (pink) was quantified using ImageJ 1.37c.

To further confirm the involvement of endosomal pathway in internalization of GPI-anchored MFI2, the SK-MEL 28 cells or S34N-transfected cells were stained for anti-TfR (OKT9), anti-MFI2 (L235) and anti-EEA1 antibodies. The cells were incubated at 37 °C for 20 min before they were fixed and labeled with secondary antibodies at 4 °C. The confocal results show that the dominant negative rab5 restricted the localization of GPI-anchored MFI2 to the periphery of the cell with no significant co-localization with EEA- 1 inside the cells, whereas in the cells with functioning rab5, there is a greater co localization between EEA- 1 and MFI2 inside the cell (Fig. 7a, b). Similar result was also observed for TfR in these cells (Fig. 7c, d). These data suggest that functioning endosomes are essential in the intracellular trafficking of GPI-anchored MFI2 in melanoma cells.

## Discussion

In the kingdom Animalia, several examples of non-Tf bound iron (NTBI) uptake have been documented [18, 65], and studies have shown that both high-affinity and low-affinity mechanisms are involved in NTBI uptake but little is known about the molecular mechanisms involved in any of these processes. These alternative iron uptake processes are energy dependent and are not regulated by the level of iron in the cell [66]. In the case of a rare genetic disorder, atransferrinemia, where patients possess little or no plasma transferrin [67], dietary iron is still absorbed (non-Tf bound form) and transported to organs, such as heart, liver, kidney and pancreas [67–69]. Furthermore, hypotransferrinemic mice show dramatic iron overload in all but hematopoietic tissues despite lower serum Tf levels [70].

**Fig. 8:**
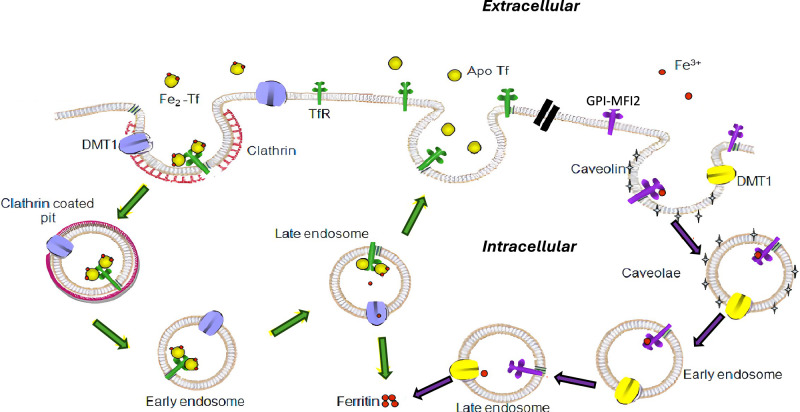
The proposed transferrin / transferrin independent model of iron uptake mediated by GPI-MFI2. GPI-MFI2 bound with ferric iron is internalized via caveolae-dependent endocytosis. As a comparison, TfR mediated iron-Tf uptake through clathrin-dependent endocytosis. The caveolae and the clathrin-coated vesicles either mature into or fuse with endosomes separately. Both proceed through the endosomal pathway where iron is released from either GPI-MFI2 or Tf due to the acidic environment within the endosomes. Iron then exits the endosome and is incorporated into cytoplasmic ferritin for storage. This model integrates our findings with current understanding of endocytic pathway organization [74, 92].

The present study provides the first stepwise description of any NTBI process, and we propose the model shown in Fig. 8 as a summary of this data. Our previous results suggest that at normal pH and salt concentrations MFI2 binds a single atom [28] and the binding affinity of MFI2 to iron is intermediate between the high and low affinity lobes of Tf [28]. This is important for understanding the relevance of NTBI as it has been demonstrated that upon binding of Tf to TfR, a conformation change occurs in Tf that hinders the release of iron from N-lobe of Tf [13] and the authors suggest that only one atom of iron is released within the cell during each round of internalization. Given that mammalian cells in culture may express approximately 1.0 × 10^6^ molecules of MFI2/cell [27], the efficiency of iron uptake via MFI2 appears to rival that of the classical Tf-TfR pathway. More recently, the functional and evolutionary relevance of GPI-MFI2 has been clearly established by the demonstration of its critical role in epithelial septate junction assembly in *Drosophila* [36].

Examining the native distribution of GPI-anchored proteins has posed a scientific challenge. Studies on the GPI-anchored folate receptor provided the first biochemical clues that caveolae could mediate uptake of molecules in a variety of cells. In MA106 cells, the folate receptor has been shown to internalize bound folate through caveolae [55]. The internalized folate dissociates from its receptor in response to an acidic environment and diffuses directly into the cytoplasm due likely to its hydrophobicity [71]. Other studies however, have shown that the internalization of GPI-anchored folate receptor is independent of caveolin expression [72]. The localization of GPI-anchored proteins after internalization is also a controversial subject. Rothberg et al. observed that the folate receptor is not found in either the endosomes nor the lysosomes, while Mayor et al. demonstrated that GPI-anchored folate receptor can be localized to the endosomes [55, 73]. The heterogeneity in GPI-protein trafficking likely reflects differences in lipid raft association, protein clustering, and cell-type-specific expression of endocytic machinery [21].

Furthermore, GPI-MFI2 represents one of the very few examples where the delivery of a ligand by GPI-anchored protein is followed during the endocytic process. We demonstrated in the present study, that GPI-MFI2 enters the SK-MEL 28 cell through caveolae (Fig. 1d), not clathrin vesicles (Fig. 1a). In our studies we do not use polyclonal secondary antibodies that could trigger a cross-linking event rather than distributing constitutively in caveolae (Fig. 2) [51]. Furthermore, previous studies have demonstrated that the redistribution event is prevented by using stronger fixing reagents of 0.3–0.5% glutaraldehyde along with 3% paraformaldehyde [51]. To avoid this potential artifact, we employed much stronger fixing reagents than suggested by Mayor et al., to exclude artefactual redistribution and we then confirmed the distribution of GPI-MFI2 to caveolae with fluorescent labeled Fab fragments made from monoclonal antibody against MFI2 that exclude the possibility that antibody cross linking triggers internalization (Fig. 2). These experiments thus, go beyond previous studies and confirm the existence of caveolae uptake. Furthermore, confirmation of GPI-MFI2 distribution to caveolae was made with cryo-immunoelectron microscopy where only direct gold conjugated primary antibodies were used (Fig. 3). Sucrose gradient sub-cellular fractionation of SK-MEL 28 cells demonstrated that iron bound GPI-MFI2 is able to fractionate as an intact unit to the same fraction as caveolae (Fig. 4). Disruption of functional caveolae vesicles by cholesterol-binding reagents (filipin and nystatin) proved to be both detrimental as the internalization of GPI-MFI2 was completely blocked (Fig. 4c). Taken together, these results show that GPI-MFI2 mediates iron uptake through the caveolae-dependent endocytic pathway.

Next, we devised experiments to address the subsequent steps in GPI-MFI2 uptake from their membrane proximal internalization into caveolae. Thus, the subsequent internalization of GPI-MFI2 and TfR to the endosomal compartment is demonstrated through a series of time course internalization experiments. GPI-MFI2 co-localized with endosomes at 30 minutes after endocytosis (Fig. 5), implying an intersection of caveolae and endosomal endocytic pathway. Disruption of this trafficking by removing the GPI-anchor from MFI2 by PI-PLC treatment resulted in the loss of intracellular transfer of iron from MFI2 to ferritin (Fig. 6). The endosome disruption via dominant negative Rab5 led to decreased cellular iron loading, which suggests the fusion and maturation of endosomal vesicles are required for the transport of iron by GPI-MFI2 to the iron storage protein, ferritin (Figs. 6,7). Furthermore, our results suggest that the trafficking of Tf/TfR complex is faster than GPI-MFI2. In support of this observation, the data of Mayor et al. also addresses this specific stage of GPI-linked protein uptake by showing that GPI-anchored folate receptor is trafficked to the endosome and that the process is three times slower than the trafficking of Tf/TfR [73]. Finally, the association of MFI2 with caveolae may be facilitated by its localization to specific lipid raft microdomains enriched in cholesterol and sphingolipids, which serve as platforms for caveolar assembly [74].

### Physiological and pathological relevance of MFI2

The physiological role of MFI2 has been debated for decades, particularly following Rahmanto and Richardson’s comprehensive studies using MFI2 knockout and overexpressing mice, which found no significant role for MFI2 in systemic iron metabolism or development under standard laboratory conditions [75, 76]. However, these findings do not necessarily negate the importance of our mechanistic discoveries. Rather, they suggest that the biological significance of MFI2 may be context-dependent and emerge under specific physiological or pathological conditions that are not recapitulated in standard mouse husbandry.

Several lines of evidence support the relevance of understanding MFI2-mediated iron transport mechanisms despite the mouse knockout phenotype. The functional redundancy is common in iron metabolism. The mammalian iron transport system includes multiple overlapping pathways (transferrin receptor 1 and 2, DMT1, ZIP14, lipocalin-2 receptor, and others), and compensation by these alternative mechanisms in knockout animals is well-documented [77, 78]. The lack of a knockout phenotype under basal conditions does not preclude important functions under stress conditions, iron overload or deficiency states, specific developmental windows, or in particular tissues. Indeed, many iron transport proteins show tissue-specific or context-dependent roles that only become apparent under challenge conditions [79].

MFI2 expression is markedly upregulated in pathological states including melanoma, glioblastoma, and Alzheimer’s disease [30, 50, 80]. This disease-associated expression pattern suggests that MFI2 may confer selective advantages to transformed or diseased cells, possibly through the caveolae-dependent iron uptake mechanism we describe. Rapidly proliferating cancer cells have elevated iron requirements to support DNA synthesis, mitochondrial function, and numerous iron-dependent enzymes [81]. Understanding this mechanism is crucial for developing therapeutic strategies targeting MFI2-expressing tumors.

The evolutionary conservation suggests functional importance. MFI2 belongs to the ancient transferrin family, with origins dating back over 670 million years [82]. The maintenance of this protein throughout vertebrate evolution despite apparent redundancy implies selective pressures that may not be captured in laboratory mouse models. Proteins that appear functionally redundant under laboratory conditions often prove essential under natural selection pressures including pathogen exposure, dietary variation, and environmental stress [83].

The *Drosophila* studies demonstrating the role of MFI2 in septate junction formation [36] indicate that this protein has acquired tissue-specific functions beyond systemic iron homeostasis. Our demonstration of a unique caveolae-dependent internalization mechanism, distinct from the clathrin-mediated pathway used by canonical transferrin receptor, provides the mechanistic basis for understanding how MFI2 might serve specialized cellular functions in specific anatomical or pathological contexts. The slower kinetics and different trafficking itinerary of caveolar versus clathrin pathways [84] could direct MFI2-bound iron to distinct intracellular compartments or enable different regulatory mechanisms compared to TfR-mediated uptake.

Thus, while MFI2 may not be essential for bulk iron metabolism under standard conditions, elucidating its cell biological mechanism is critical for understanding: (1) its role in disease states where it is highly expressed, (2) potential compensatory mechanisms in tissue-specific or stress contexts, (3) evolutionary pressures that have maintained this apparently redundant pathway, and (4) opportunities for therapeutic intervention in MFI2-expressing cancers.

### Implications for cancer biology and therapy

Historically, the role of GPI-MFI2 as an iron transporter has been questioned due to the absence of an iron regulatory element (IRE), suggesting that its expression is not directly regulated by intracellular iron levels [85]. However, these critiques overlook that neither TfR-2 nor transferrin itself possesses IREs [86]. Studies have demonstrated iron binding and uptake by GPI-MFI2 in *Drosophila*, reaffirming our initial findings regarding iron uptake via GPI-MFI2. Expanding upon previous work in non-transferrin-bound iron (NTBI) transport, our current studies reveal that GPI-MFI2 can effectively donate iron to ferritin (Fig. 6). Treatment with PI-PLC on GPI-MFI2-transfected TfR-deficient TRVb cells significantly reduces the amount of iron transferred to ferritin. Notably, untransfected TfR-deficient TRVb cells, lacking functional TfR, can still accumulate some iron in ferritin (Fig. 6), presumably through NTBI pathways. This suggests the presence of NTBI in untransfected cells that can bind to ^55^Fe-NTA and is sensitive to PI-PLC treatment. While it has been proposed that this PI-PLC sensitive component could be GPI-anchored hamster MFI2 [27], further investigation is warranted to establish this connection definitively.

Our findings have important implications for cancer biology and therapy. MFI2 is highly expressed in melanoma and other malignancies [4, 38, 50], and Smith et al. (2006) previously suggested that MFI2-caveolin-1 interaction might contribute to resistance against MFI2-targeted antibody-drug conjugates [32]. Our mechanistic characterization of caveolae-dependent MFI2 internalization provides molecular insights into this resistance mechanism. Specifically, the slower kinetics of caveolar internalization compared to clathrin-mediated endocytosis (as demonstrated in our comparison with transferrin receptor in Fig. 5) may reduce the efficiency of drug delivery for MFI2-targeted therapeutics. Additionally, differential trafficking through caveolar pathways versus classical endosomal compartments may alter the intracellular fate of antibody-drug conjugates, potentially directing them away from degradative lysosomes where cytotoxic payloads are typically released [87].

Furthermore, understanding the caveolae-dependence of MFI2 function suggests novel therapeutic strategies. Inhibitors of caveolar endocytosis could potentially selectively target MFI2-dependent iron uptake in tumors with high MFI2 expression while sparing normal cells that primarily use transferrin receptor-mediated iron acquisition. This selectivity could be therapeutically exploited to create an “iron addiction” vulnerability in MFI2-high tumors. Iron chelation therapy has shown promise in cancer treatment [88], and combining iron chelation with caveolar inhibition might synergistically target MFI2-expressing malignancies. Furthermore, exploiting the caveolar pathway for drug delivery specifically to MFI2-high tumors represents an alternative approach to antibody-drug conjugates that could circumvent the resistance mechanisms we have identified.

The high expression of MFI2 in melanoma cells (including the SK-MEL 28 cell line used in our study, which exhibits some of the highest MFI2 levels described to date [34]) may reflect an adaptation that provides proliferative advantages under the specific metabolic demands and microenvironmental conditions of tumors. The hypoxic, nutrient-limited tumor microenvironment creates intense competition for iron [89], and MFI2 may provide an alternative iron acquisition route that complements TfR when transferrin-bound iron becomes limiting. The caveolae-dependent pathway we describe may be particularly well-suited to function under these stressed conditions, as caveolae have been implicated in mechanosensing, membrane repair, and stress responses [90].

In this context, fascinating evidence indicates that MFI2 may function as a tumor suppressor in malignant melanoma, contrary to its previously proposed tumor-promoting role [33]. CRISPR/Cas9 knockout of MFI2 enhanced melanoma cell invasiveness and altered expression of genes linked to tumor progression, while in vivo studies in SCID mice showed increased tumor growth and metastasis in MFI2-deficient cells. Patient dataset analyses further revealed a positive correlation between MFI2 expression and overall survival, highlighting its potential clinical relevance. Complementary studies, including the antitumor efficacy of the MFI2-targeted ADC SGN-CD228A and reviews of its broader physiological roles, support this emerging perspective [35]. Collectively, these findings challenge conventional views of MFI2, and our data reinforce its context-dependent tumor-suppressive role, underscoring the need for further mechanistic studies.

Overall, the data from this study extend the understanding of iron uptake mechanisms in mammals, specifically highlighting the role of caveolae in NTBI uptake [18]. Our findings suggest that GPI-MFI2 serves as an iron-binding receptor facilitating NTBI uptake through a caveolae-dependent pathway. This mechanism allows iron to be internalized and released within the endosomal environment before being transferred to the cytoplasm for ferritin storage, delineating a non-transferrin iron route that appears to be evolutionarily conserved.

Our findings introduce the caveolae-mediated pathway as an additional, regulated NTBI transport mechanism, which could play a key role in specific tissues and under iron overload conditions. This insight enhances our understanding of iron’s systemic management and may reveal potential therapeutic targets for iron dysregulation diseases, such as neurodegeneration and metabolic disorders. These results thus lay the groundwork for future studies investigating the diverse physiological functions of caveolae and GPI-MFI2 in iron transport, as well as their evolutionary conservation across animal species.

## Material and methods

### Tissue culture, cell, and constructs

The human melanoma cell line SK-MEL 28 was obtained from American Tissue Culture Collection (ATCC, Manassas, VA) and maintained in Dulbecco’s modified Eagle’s medium (DMEM) (Invitrogen Life Technologies, Burlington, ON) supplemented with 10% (v/v) heat-inactivated fetal bovine serum (FBS) (Invitrogen Life Technologies, Burlington, ON), 2 mM glutamine and 20 mM Hepes. This cell line was selected based on its exceptionally high expression of melanotransferrin (MFI2/CD228) as previously documented [34, 38], making it an optimal system for studying MFI2-specific trafficking mechanisms without confounding background signals from the abundant TfR pathway. TRVb cell line, a Chinese Hamster Ovary (CHO) cell line that does not express functional endogenous hamster transferrin receptor [91], was obtained from Dr. F. Maxfield (New York University, NY). It was maintained in Ham’s F12 media (Invitrogen life technologies Inc., Burlington, ON) supplemented with 10% (v/v) heat-inactivated fetal bovine serum (FBS) (Invitrogen Life Technologies, Burlington, ON), 2 mM glutamine and 20 mM Hepes. All cells were maintained at 37 °C in a 5% CO_2_ humidified incubator. The dominant negative Rab5 (pEGFP-Rab5-S34N) construct was generous gift from Dr. Robert E. Lodge (National Institutes of Health).

### Antibodies

Primary antibodies (Abs) used were as follow: mouse anti-human TfR Ab (OKT-9; ATCC, Manassas, VA), mouse anti-human MFI2 Ab (L235; ATCC, Manassas, VA), rabbit anti-human ferritin L-chain (ferritin; DAKO, Carpinteria, CA), rabbit anti-human caveolin-1 (cav-1; BD Transduction Laboratories, Franklin Lakes, NJ), rabbit anti-human clathrin heavy chain (Cla-HC; Santa Cruz Biotechnology Inc., Santa Cruz, CA), mouse anti-human adaptin-β (β-adaptin; BD transduction laboratories, Franklin lakes, NJ), goat anti-human early endosome marker-1 (EEA-1; Santa Cruz Biotechnology Inc., Santa Cruz, CA), Alexa 488-conjugated goat Ab (Molecular Probes Inc., Eugene, OR), Alexa 568-conjugated goat Ab (Molecular Probes Inc., Eugene, OR) and Cy5-conjugated rabbit Ab (Jackson Laboratories, West Grove, PA). Monoclonal L235 Fab antibody fragment was made by digestion of mouse anti-human MFI2 Ab with papain (Sigma-Aldrich, Oakville, ON), and the resulting Fab fragment was directly conjugated with Cy5.

### Sucrose gradient sub-cellular fractionation and western blot

Cells were lysed on ice in 1% (v/v) Triton X-100 in 140 mM KCl, 10 mM Tris-Cl, pH 7.5. 80% (w/v) sucrose was added to the lysate to make a 40% sucrose lysate mixture. The lysate mixture was overlaid with 30% cold sucrose followed by 5% sucrose. This gradient was then centrifuged for 16 h at 4 °C. Eight fractions were collected and each was subjected to 10% (w/v) SDS-PAGE and transferred to Immobilon membranes (Biorad) by electroblotting. The membrane was incubated with the primary Ab and then with horseradish peroxidase-conjugated secondary Ab. The ECL Western blotting detection system (Amersham Pharmacia Biotech) was used to detect the chemiluminescence.

### Immunoprecipitation

SK-MEL 28 cells were washed three times in Iscove’s modified Dulbecco media (IMDM) (Invitrogen) for 1 hour each time at 37 °C to remove endogenous iron and serum transferrin. The cells were then incubated with ^55^Fe-NTA at 37 °C, followed by cell lysis in 1% (v/v) Nonidet-P40 (NP-40) (Sigma) in lysis buffer (20 mM Tris-Cl pH 7.4, 150 mM NaCl, 2 mM EDTA and 1 tablet of protease inhibitor cocktail (Roche) on ice). The lysate was collected and centrifuged at 11,000 rpm for 15 min at 4 °C. The supernatant was collected and pre-cleared with 3 µl of normal rabbit serum for an hour and another subsequent hour with 30 µl of protein G sepharose bead slurry (Amersham Biosciences, Arlington Height, IL) under agitation. The lysate was immunoprecipitated with antibodies against human MFI2 and ferritin for two hours at 4 °C, followed by the addition of pre-washed protein G sepharose bead slurry (30 µl) for an hour at 4 °C. The sample was washed twice in buffer B (0.2% NP-40, 10 mM Tris-Cl pH 7.5, 150 mM NaCl, 2 mM EDTA), once in buffer C (0.2% NP-40, 10 mM Tris-Cl pH 7.5, 500 mM NaCl, 2 mM EDTA) and once in buffer D (10 mM Tris-Cl pH 7.5). Finally, the sample was resuspended in PBS before transferred to scintillation vials along with the ReadySafe scintillation counting fluid (Beckman Colter Inc., Fullerton, CA). The radioactivity associated with the immunoprecipitate was measured with a Beckman LS6000IS Liquid Scintillation counter.

### Immunofluorescent confocal microscopy

SK-MEL 28 cells were grown on sterile glass coverslips. Cells were labeled with primary Abs against human MFI2 (L235), or anti-human transferrin receptor (OKT9) or with Cy5 conjugated anti-human MFI2 Fab fragment for 1 h at 4 °C. Cells were then incubated at 37 °C for various time points to allow endocytosis to take place. Following incubation, cells were fixed in 4% paraformaldehyde and 2% glutaraldehyde (Sigma) in PBS for 30 min at room temperature. Cells were permeabilized with 0.1% saponin in 1% BSA/PBS for 20 min at room temperature. Cells were also stained with a second set of primary antibodies against various vesicles for 30 min at room temperature: rabbit anti-human caveolin-1, goat anti-human clathrin, and goat anti-human early endosome antigen 1 (EEA1). Alexa Fluors^®^ (Molecular Probes) were used to visualize the staining patterns. After staining, cells were incubated in Slow Fade (Molecular Probes) equilibration buffer and mounted in Slow Fade glycerol solution. The fluorescent cell images were captured using BioRad Radiance Plus confocal laser scanning microscope and Nikon TE2000 inverted microscope with EZ-C1 software version 3.0. Data analysis was performed with ImageJ 1 and Adobe Photoshop CS version 8.0.

### Cryoimmunoelectron microscopy

SK-MEL 28 cells grown on glass coverslips were labeled with 5 nm gold conjugated primary antibody against either MFI2 or transferrin receptor at 37 °C to allow for internalization. Excess antibodies were removed and the cells were fixed in 4% paraformaldehyde and 2% glutaraldehyde for 30 min. The cells were then permeabilized before being labeled with 10 nm gold conjugated anti-caveolin or 10 nm gold conjugated anti-clathrin antibody for 30 min at 4 °C. Excess antibodies were removed before the cells were fixed with 1% osmium tetroxide in 0.1 M phosphate buffer (Canenmco) in the microwave (Microwave fixation: 2 min on 100 W under vacuum, 2 min off under vacuum and 2 min on 100 W under vacuum). This was repeated and the samples were rinsed twice in distilled water. The cells were removed from the cover slips before the dehydration protocol in the microwave. Sequential dehydrations in ethanol (50%, 70%, 95%, 100%, 100%, and 100%) were carried out at 200 W for 40 s, with a gentle centrifugation between each step. The cells were pelleted and infiltrated with 1:1 acetone:resin in the microwave at 300 W under vacuum. The resin was a 1:1 mixture of EPON and Spurr’s soft mixture. The cells were infiltrated three times with 100% resin (same as above, fresh resin each infiltration) and baked at 65 °C overnight. The blocks were cut using a diatome diamond knife and a Leica Ultracut T ultramicrotome and stained with 2% uranyl acetate and finally with Reynold’s lead citrate for 30 and 15 min, respectively. The sections were viewed on a Hitachi H7600 TEM and digital pictures taken using an AMT camera built into the H7600.

### Statistical analysis

In the experiments, error bars represent either the standard deviation (SD) of three separate experiments or the standard error of the mean (SEM), as indicated in the figure legend. Statistical significance was assessed using appropriate tests as indicated in figure legends. For comparing proportions of GPI-MFI2 and TfR colocalization to caveolae and clathrin coated pits, the z statistic was calculated to compare two proportions. For other comparisons, Student’s t-test (two-tailed, unpaired) or one-way ANOVA with appropriate post-hoc tests were used as indicated. *p* < 0.05 was considered statistically significant. Sample sizes (n) represent independent biological replicates and are indicated in figure legends.

## Supplementary information

Supplemental Data-Xray Film Blots Fractionation

## Data Availability

All data supporting the findings of this study are available within the paper and its supplementary information files. Raw microscopy images, flow cytometry files, scintillation counting data, and Western blot images are available from the corresponding author upon reasonable request. Plasmid constructs are available through material transfer agreements with the original providers (Dr. Ari Helenius for caveolin constructs; Dr. Robert E. Lodge for Rab5 constructs).
