# Supplementary material for: Lipid-anchored melanotransferrin mediates transferrin-independent iron uptake and ferritin storage in mammals
Source: Cell Death Discov. 2026 Apr 17;12:253. doi: 10.1038/s41420-026-03043-9 (PMC13213057; doi:10.1038/s41420-026-03043-9)

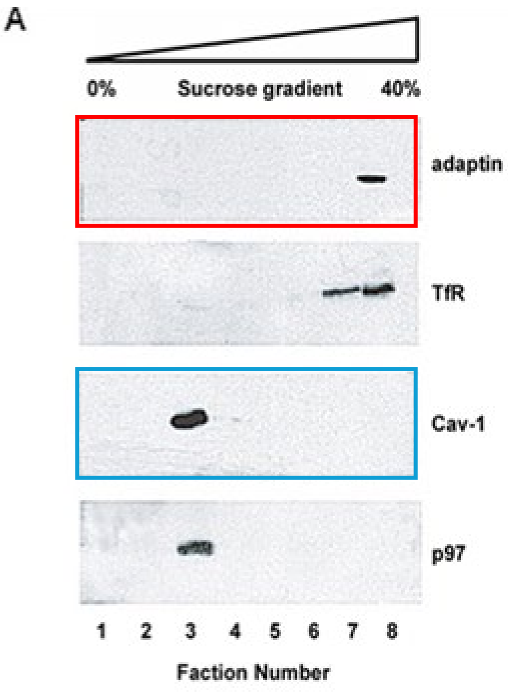

Original X-ray Films for Blots for Figure 5A: Adaptin and Caveolin

Adaptin and Caveolin – original blot image

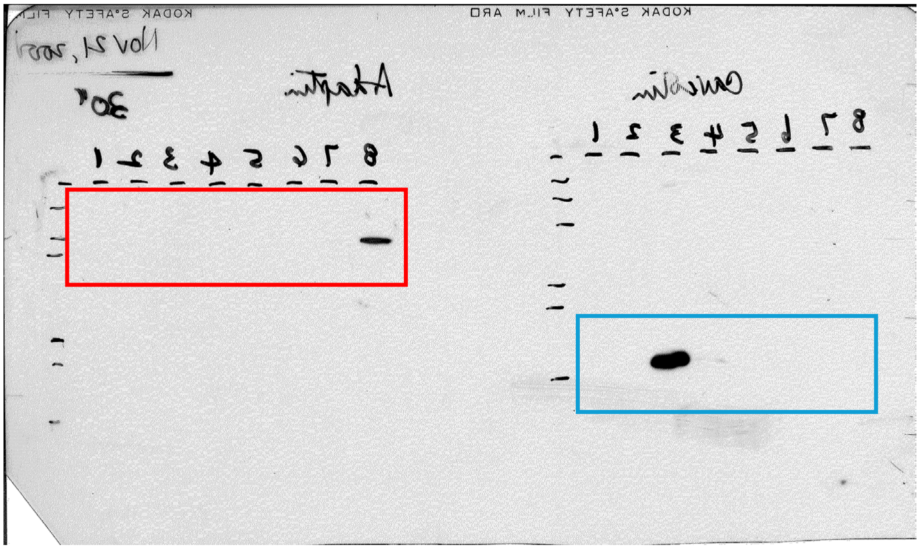

Adaptin and Caveolin - film flipped

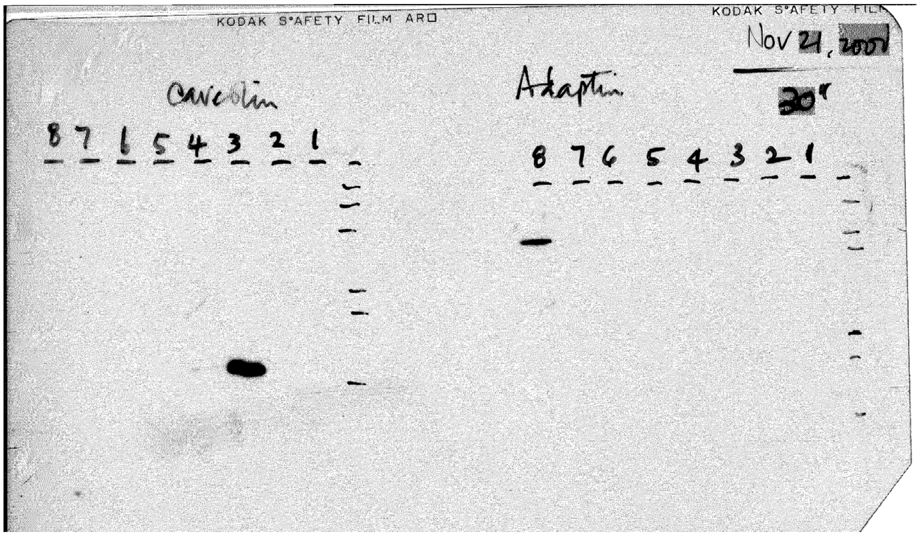

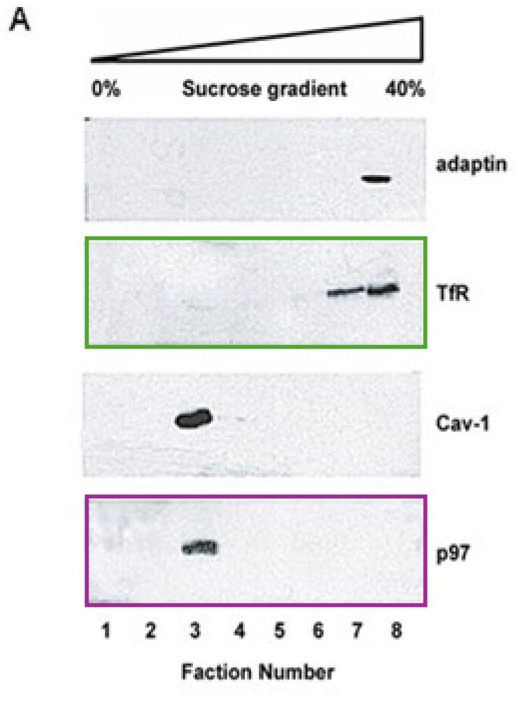

Original X-ray Films for Blots for Figure 5A: TfR and p97

Transferrin Receptor

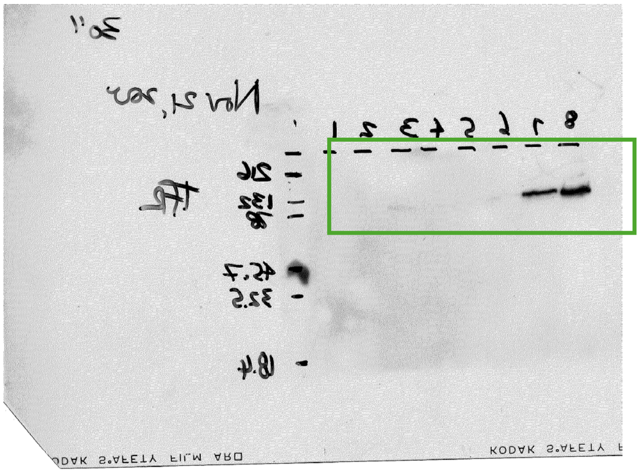

p97 (Melanotransferrin)

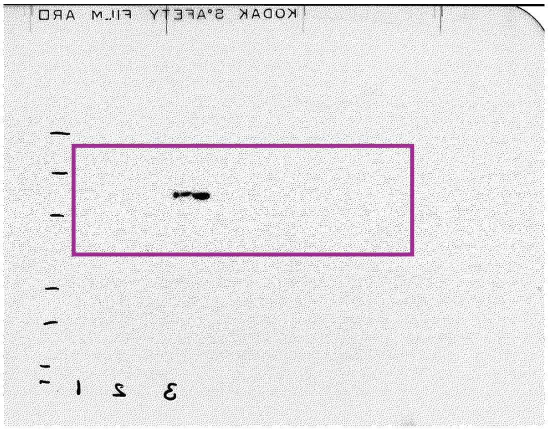

Transferrin Receptor – film flipped

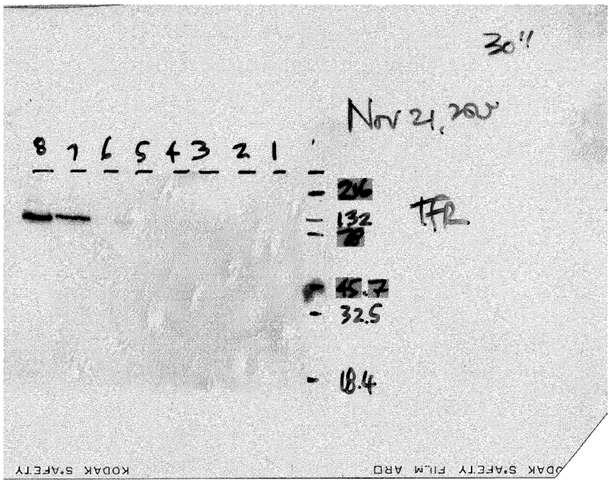

p97 (Melanotransferrin)- film flipped

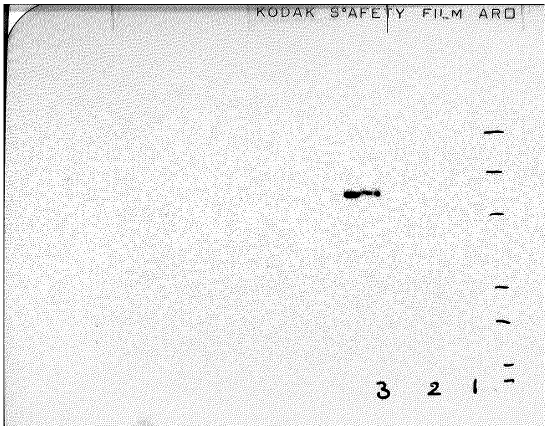

Supplement: Supplementary file 1 — Supplemental Data-Xray Film Blots Fractionation [file 41420_2026_3043_MOESM1_ESM.pdf]
